# Utilizing Nanoparticles of Hesperidin Loaded on Layered Double Hydroxide to Reduce Hepatotoxicity Caused by Paracetamol in Rats: Controlling of Biotransformation, Oxidative Stress, Inflammation, and Apoptosis

**DOI:** 10.3390/pharmaceutics17040429

**Published:** 2025-03-27

**Authors:** Deyaa A. Shaban, Ahmed A. G. El-Shahawy, Mohamed I. Zanaty, Zienab E. Eldin, Mohamed Abd-Elbaset, Anwar Shams, Shadi Tamur, Osama M. Ahmed

**Affiliations:** 1 Department of Biotechnology and Life Sciences, Faculty of Postgraduate Studies for Advanced Sciences (PSAS), Beni-Suef University, Beni-Suef 62511, Egypt; deyaa4ahmed@yahoo.com (D.A.S.); zanaty012@yahoo.com (M.I.Z.); 2 Materials Science and Nanotechnology Department, Faculty of Postgraduate Studies for Advanced Sciences (PSAS), Beni-Suef University, Beni-Suef 62511, Egypt; ahmed.elshahawy@psas.bsu.edu.eg (A.A.G.E.-S.); t-zienabessam@zewailcity.edu.eg (Z.E.E.); 3 Department of Pharmacology and Toxicology, Faculty of Pharmacy, El Salehya El Gadida University (SGU), El Salheya 44813, Egypt; 4 Department of Pharmacology, College of Medicine, Taif University, P.O. Box 11099, Taif 21944, Saudi Arabia; 5 Research Center for Health Sciences, Deanship of Graduate Studies and Scientific Research, Taif University, Taif 26432, Saudi Arabia; 6 High Altitude Research Center, Taif University, P.O. Box 11099, Taif 21944, Saudi Arabia; 7 Department of Pediatric, College of Medicine, Taif University, P.O. Box 11099, Taif 21944, Saudi Arabia; shaditamur@tu.edu.sa; 8 Physiology Division, Zoology Department, Faculty of Science, Beni-Suef University, Beni-Suef 62521, Egypt; osama.ahmed@science.bsu.edu.eg

**Keywords:** paracetamol, hesperidin nanoparticles, hepatotoxicity, layered double hydroxide

## Abstract

**Background/Objectives**: The most used antipyretic and pain relief treatment is paracetamol (acetaminophen), also known as N-acetyl-para-aminophenol (APAP). However, it is considered potentially hazardous if consumed repeatedly in large doses or over prolonged periods. This investigation explores the effectiveness of hesperidin (Hesp) and Hesp loaded on layered double hydroxide nanoparticles (Hesp-NPs) in inhibiting the progression of acute hepatotoxicity in rats induced by APAP. **Methods**: LDH-Hesp-NPs were prepared and characterized. Male Wistar rats were orally treated with Hesp and Hesp-NPs at the same adjusted dose (100 mg/kg) every other day for six weeks. After 2 h of the first doses of Hesp and Hesp-NPs, the rats received one oral dose of APAP (750 mg/kg). **Results**: Administering of Hesp and Hesp-NPs to APAP-treated rats significantly reduced oxidant parameter (malondialdehyde) and serum enzymes (ALT, AST, LDH, and ALP) associated with liver function. Antioxidant markers in the liver, such as catalase and glutathione, also increased notably. Moreover, Hesp and Hesp-NPs enhanced the mRNA expression of liver *UGT1A6*, *IL-10*, and *HO-1*. Conversely, the mRNA expressions of liver *CYP1A1*, *KEAP1*, *TGF-β*, *P53*, and *BAX* decreased. These improvements in biochemical and molecular markers were corroborated by liver histopathology. **Conclusions**: Hesp and Hesp-NPs protect significantly against APAP-induced hepatotoxicity in male Wistar rats. Hesp-NPs treatment was more potent. The protective effects may be mediated *via* modulation of APAP biotransformation, oxidative stress, inflammation and apoptosis.

## 1. Introduction

An essential component of the body, the liver aids in the fight against infections as well as crucial biochemical and physiological processes like homeostasis, development, energy production, nutrition delivery, and drug and xenobiotic detoxification [1]. Fifty percent of acute liver failure cases and five percent of hospital admissions are related to drug-induced liver injury. Hepatotoxicity refers to liver damage brought on by drugs and other chemicals, which induce oxidative stress. Oxidative stress is brought on by a decrease in the effectiveness of antioxidant defenses and an increase in the production of reactive oxygen species and other reactive intermediates is a main concern in hepatotoxicity [2].

Acetaminophen (APAP), frequently referred to as paracetamol, is an essential factor in drug-related morbidity and mortality in humans, as even a single hazardous dose may end up in substantial liver necrosis. Although studies have demonstrated that APAP may be hepatotoxic at doses less than 4 g per day, the drug is generally regarded as safe at therapeutic doses [3]. Cytochrome P4502E1 metabolically activates paracetamol to produce the reactive metabolite N-acetyl-p-benzoquinoneimine (NAPQI). In turn, NAPQI conjugates with glutathione (GSH) to undergo detoxification. GSH is, therefore, the first line of defense against the production of free radicals caused by paracetamol. Total hepatic GSH was shown to be reduced in paracetamol poisoning as a result of hepatic cell destruction. Consequently, there is less production of the NAPQI glutathione conjugate [4].

There has recently been an interest in researching natural substances that have the ability to scavenge free radicals [5]. Flavonoids are a class of phenolic substances commonly found in plants. A large number of flavonoids were studied in their free state and as glycosides [5,6,7]. Hesperidin (Hesp) is a flavanone glycoside, and it is one type of bioflavonoid natural product within citrus fruits, including oranges, lemons, and grapefruits, with potent pharmacological characteristics, such as anti-inflammatory, antihypertensive, anti-edema, anti-arthritic, and anti-atherogenic [5]. Hesp has a wide range of applications in the defense against ailments such as cardiovascular and neurodegenerative diseases. Further, it interacts with cellular targets, preventing the proliferation of cancer cells via cell cycle arrest [8].

Even while Hesp is safe and effective, it has an array of significant disadvantages. Due to its relatively limited oral bioavailability and water solubility, it has not been used clinically. A number of tactics have been created, such as the creation of efficient delivery systems with Hesp-like drugs [9]. A potential solution to this problem is the use of nanosized drug carriers [10]. Layered double hydroxides (LDHs) are prospective targeted carriers for the delivery of various medicines with poorly soluble or limited half-life. Natural products can intercalate bioactive compounds via anion exchange [11]. Yet, the reduction in free radical production caused by Hesp administration in rats given APAP may indicate Hesp’s anti-inflammatory and antioxidant capacities in the context of acute inflammation [12]. In nicotinamide/streptozotocin-induced diabetic rats, Hesp packed on MgAl-layered double hydroxide (LDH) nanocomposite was previously tested to assess its anti-inflammatory, antioxidant, and antidiabetic properties [13].

Given the foregoing, the current study was split into two sections: the first section dealt with the preparation and characterization of Mg-Al-LDH and a nanoformula consisting of Mg-Al-LDH loaded with Hesp nanoparticles (NPs). We called this formula Hesp-NPs. Examining the possible effectiveness of Hesp in its native form and Hesp-NPs to postpone the development of rat hepatic damage produced by paracetamol is the main question of the second section.

## 2. Materials and Methods

### 2.1. Materials and Agents

**Scheme 1:**
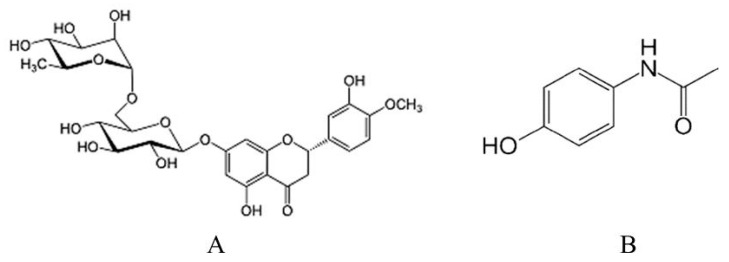
Chemical structure of Hesp (**A**) and APAP (**B**).

Chemical agents that involve magnesium nitrate (Mg(NO_3_)_2_.6H_2_O), aluminum nitrate (Al (NO_3_)_3_.9H_2_O), sodium hydroxide (NaOH), and hydrochloric acid (HCl) were provided via Trade Chemicals and Medical Equipment El Gomhouria Company (Cairo, Egypt). Hesp (Scheme 1A) and paracetamol (APAP) (Scheme 1B) were provided by Sigma Chemicals Co., St. Louis, MO, USA.

Aspartate transaminase (AST), alanine transaminase (ALT), lactate dehydrogenase (LD), alkaline phosphatase (ALP), albumin, and bilirubin kits were obtained from Human Diagnostics Worldwide, Ahrensburg, Germany. Rat tumor necrosis factor-alpha (TNF-α) ELISA kit (Catalog Number. CSB-E11987r) was obtained from Cusabio, Houston, TX, USA. Rat alpha-fetoprotein (AFP) ELISA kit was purchased from BT LAB, Jiaxing, Zhejiang, China. The malondialdehyde (MDA) measurement kit (M3637) was obtained from Tokyo Chemical Industry Co., Ltd., Tokyo, Japan. The glutathione kit (Product Code: T419) was obtained from Dojindo Laboratories (Kumamoto, Japan). The catalase kit (Cat. No: KBF033) was delivered from KRISHGEN BioSystems, Whittier, CA, USA. Rat Interleukin 4 (IL-4) Quantikine ELISA Kit (Catalog no. R1000) was supplied by R&D Systems, Inc. (Minneapolis, MN, USA). Every other chemical was acquired from a typical commercial supplier and was of analytical grade.

### 2.2. Preparation of Mg AL-LDH and Mg AL-LDH-Hesp

The synthesis of LDH and drug-LDH composite materials was prepared according to El-Shahawy et al. [13]. A total of 100 mL solution, including (0.045 M) Mg (NO_3_)_2_.6H_2_O and (0.015 M) Al (NO_3_)_3_.9H_2_O, was dropped in (0.15 M) NaOH dropwise with rigorous stirring. Until completely precipitated at pH 8.5. The precipitation process took around 20 h, with the temperature of the precipitation being maintained at 65 °C. After filtering, the product was centrifuged and cleaned with distilled water, and it was baked for 48 h at 40 °C to dry it out. The same procedures were followed to create MgAl-LDH-Hesp nanocomposites, but 30 mg of Hesp was added to the medium prior to precipitation. After 20 h of stirring at 50 m°C, the MgAl-LDH-Hesp precipitate suspension was filtered, cleaned, and dried at 40 n°C.

### 2.3. Characterization

#### 2.3.1. Fourier Transform Infrared (FTIR) Spectroscopy

**Figure 1:**
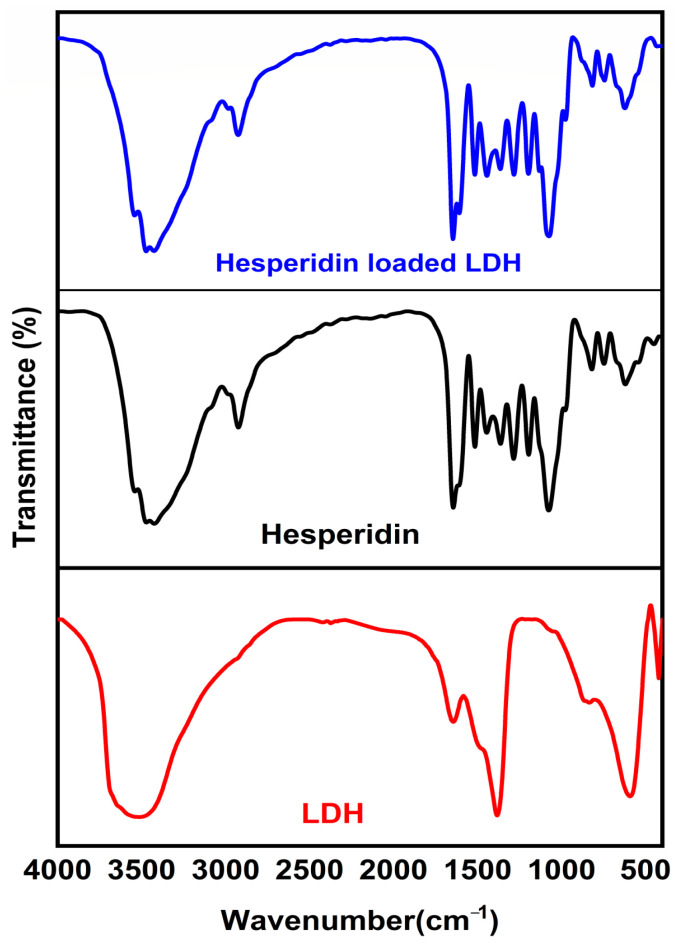
MgAl LDH, MgAl LDH–hesperidin, and hesperidin powder FTIR spectroscopy.

Using a Bruker Vertex 70 FTIRFT Raman spectrometer, the nanocomposites’ FTIR spectra were acquired with a precision of 1 cm^−1^ across a 500–4500 cm^−1^ range (Figure 1).

#### 2.3.2. Morphology

**Figure 2:**
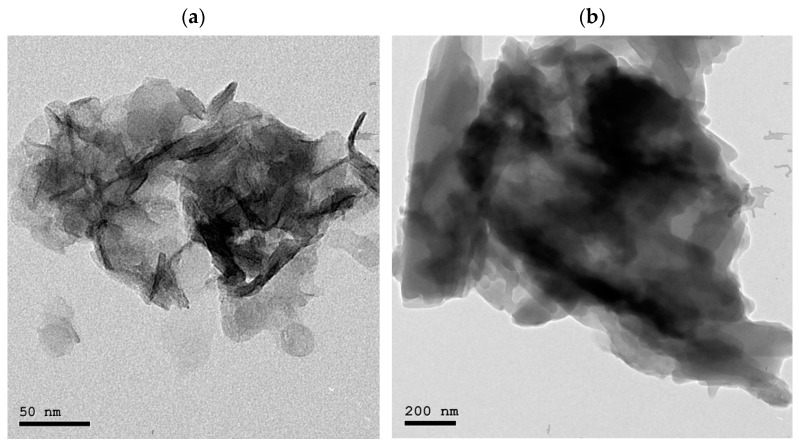
HRTEM micrographs of (**a**) MgAl LDH (**b**) MgAl LDH–hesperidin nanopowder.

High-resolution transmission electron microscopy (HR-TEM) (JEM 1400, Tokyo, Japan) was used to capture transmission electron micrographs of MgAl-LDH nanoparticles and MgAl-LDH-Hesp with an acceleration voltage of 300 KV (Figure 2).

#### 2.3.3. Particle Size and Zeta Potential Analysis

Using dynamic light scattering (DLS) and a ZS90 Zeta sizer instrument (Malvern, UK), the hydrodynamic size of Hesp incorporated MgAl-LDH in the aqueous solution was determined.

#### 2.3.4. Entrapment Efficiency (EE) and Drug Loading (DL)

By separating the supernatant containing free Hesp after centrifuging the samples for 30 min at 10,000 rpm and 4 °C, the entrapment efficiency of Hesp-loaded MgAl-LDH nanoparticles was ascertained. Using HPLC analysis, the amount of Hesp trapped in LDH was determined. Every measurement was performed three times (n = 3) [14]. This is how the Hesp entrapment efficiency (EE%) was determined:

Hesp (EE) = Drug amount taken initially − Drug amount in supernatant/Drug amount taken initially × 100(1)

Hesp (DL) = Amount of drug originally taken/amount of NPs loaded drug × 100(2)

#### 2.3.5. In Vitro Drug Release Studies

Using Lee et al.’s approach [15], the amount of Hesp released from MgAl-LDH-Hesp nanostructures in phosphate-buffered saline (PBS) at 37 °C was quantified over the course of 24 h. For varying amounts of time, Hesp-loaded LDH nanostructures were stored in a dialysis bag (MWCO: 12 kDa) filled with a PBS solution. The concentration of Hesp released from the LDH nanostructures was then determined via HPLC analysis.

### 2.4. Experimental Design

Forty male Wistar rats were used in this investigation. They weighed between 160 and 200 g. The rats had free access to water and were fed a commercial pellet meal. The rats were kept in hygienic, roomy macro-lane cages under typical laboratory settings and were divided into four groups of ten rats each. Beni Suef University’s Institutional Animal Care and Use Committee (BSU-IACUC) (Ethical Approval Number: 022-518) governed all experimental protocols. The groups were designed as follows: In group I (normal), rats received an oral dose of 1% carboxymethyl cellulose (CMC, 5 mL/kg b.w.) [16] every other day for six weeks. The rats of group II (APAP) received just one dose of APAP suspension (750 mg/kg b.w.) [17] and were given an oral dose of 1% CMC (5 mL/kg b.w.) every other day for six weeks. The group III (Hesp+APAP) rats received, every other day for six weeks, an oral dose of Hesp (100 mg/kg b.w.) [18] dissolved in 5 mL 1% CMC. After 2 h of the 1st dose of Hesp, the rats received one dose of APAP suspension (750 mg/kg b.w.). The group IV (Hesp-NPs+APAP) rats received an oral modulated dose of Hesp-NPs (100 mg/kg b.w.) dissolved in 5 mL 1% CMC every other day for six weeks. After 2 h of the 1st Hesp-NPs, the rats received one dose of APAP suspension (750 mg/kg b.w.t.).

### 2.5. Blood Sampling and Serum Analysis

The animals were sacrificed at the end of the experiment while under light diethyl ether anesthesia. The jugular vein was used to draw blood, which was then placed in gel and clot activator tubes. Centrifugation was used to separate the sera at 3000 rpm. The sera were frozen at −20 °C pending biochemical examination. Following the manufacturer’s instructions, conventional laboratory kits were used to measure the liver function parameters, including ALT [19], AST [19], ALP [20], LDH [21], albumin [22], and total bilirubin [23].

### 2.6. Liver Sampling and Analysis

The liver from each rat was quickly excised after dissection. One part of the liver (0.5 mm^3^) was fixed in neutral buffered formalin for histological investigation and processing for staining with hematoxylin and eosin according to the method of Bancroft and Stevens [24]. The stained liver sections were examined for the detection of histopathological scores, which were graded as absent (0), mild (I), moderate (II), and severe (III). Two other parts of each rat’s liver were separated and kept at −30 °C. While the second half was employed for molecular analysis and gene expression, the first part was used for the determination of markers of oxidative stress and antioxidant defense systems. Using a Teflon homogenizer (Glas-Col, Terre Haute, USA), 0.5 g of the frozen liver was homogenized in 5 mL of 0.9% sterilized sodium chloride (NaCl) (10% *w*/*v*) for the ensuing determination of determination of oxidative stress and antioxidant defense system biomarkers. Thiobarbituric acid-reactive substances (TBARS) and MDA were measured to assess lipid peroxidation in the liver [25]. The protocols of Claiborne [26] and Beutler et al. [27], were followed for measuring the liver enzyme activity of CAT and GSH, respectively. Serum TNF-α [28], AFP [29], and IL-4 [30] were measured using rat ELISA kits.

Considering molecular analysis by semi-quantitative-PCR, the gene expression of hepatic *BCL2*-associated X (*BAX*), B-cell lymphoma 2 (*BCL2*), tumor protein 53 (*P53*), Kelch-like ECH-associated protein-1 *(KEAP-1*), cytochrome P450 1A1 (*CYP1A1*), uridine diphosphate glucuronosyltransferase 1A1 (*UGT1A6*), NAD(P)H quinone oxireductase-1 (*NQO1*), NAD(P)H quinone oxireductase-2 (*NQO2*), Heme oxygenase 1 (*HO-1*), transforming growth factor-β (*TGF-β*) and interleukin 10 (*IL-10*) were determined. Using the TRIzol^®^ Reagent (Zymo Research, Tustin, CA, USA), total RNA was extracted from rat liver tissue. The COSMO PCR Master MixTM Kit (Willowfort. Co., Ltd., Nottingham, Nottinghamshire, UK) was then used for reverse transcription of the extracted RNA, following the manufacturer’s instructions. Reverse transcription-polymerase chain reaction (RT-PCR) was applied to measure the target gene expressions using the particular primers (Table 1). The PCR results were examined on a 1% agarose gel stained with ethidium bromide, and the picture was captured using the Bio-Rad Gel Doc XR System for gel imaging. ImageJ (1.51 d) was used to measure the gray scale value in order to quantify the relative expression level. Every sample and gene was normalized using the housekeeping gene β-actin [31].

### 2.7. Statistical Analysis

The data were statistically analyzed using the Statistical Package for the Social Sciences (IBM SPSS for Windows 7, version 20; SPSS Inc., Chicago, IL, USA). One-way analysis of variance (ANOVA) was utilized for statistical analysis, and Tukey’s test was used for post hoc analysis. However, *p* < 0.05 was seen as statistically significant, and *p* < 0.05 was regarded as statistically non-significant.

## 3. Results

### 3.1. FTIR Analysis

Figure 1 displays the FTIR spectra of Mg LDH–Hesp, Hesp, and pure LDH. The FTIR spectrum of pristine Mg-Al LDH exhibits characteristic bands confirming its layered double hydroxide structure. The broad O-H stretching band at 3445 cm^−1^ corresponds to hydroxyl groups and interlayer water molecules, while the H-O-H bending vibration at 1630 cm^−1^ indicates the presence of interlayer water. A strong absorption band at 1360 cm^−1^ corresponds to the carbonate (CO_3_^2−^) ions, which act as charge-balancing anions in the interlayer space. Below 1000 cm^−1^, the spectrum shows strong metal–oxygen (M-O) and metal–hydroxide (M-OH) vibrations, characteristic of Mg-O, Al-O, and Mg-OH stretching and bending modes, confirming the integrity of the LDH structure [40]. In contrast, the FTIR spectrum of pure hesperidin displays a broad O-H stretching vibration at 3408 cm^−1^, indicating hydroxyl (-OH) groups involved in intramolecular hydrogen bonding. The C=O stretching band at 1655 cm^−1^ confirms the presence of a conjugated carbonyl functional group, while the C-O-C ether stretching vibrations at 1275 cm^−1^ and 1070 cm^−1^ are characteristic of glycosidic linkages. The aromatic C=C stretching vibrations appear between 1600 and 1500 cm^−1^, and the C-H stretching vibrations are found at 3100–3000 cm^−1^ for aromatic C-H and at 2958 cm^−1^, 2925 cm^−1^, and 2878 cm^−1^ for aliphatic C-H. The C-H bending vibrations appear at 823 cm^−1^ (out-of-plane) and between 1300 and 1000 cm^−1^ (in-plane), confirming the presence of flavonoid rings. The Hesp IR spectrum was agreed with in the literature [10]

Upon loading hesperidin onto MgAl LDH, significant spectral shifts confirm successful intercalation and interaction between the drug and the LDH matrix. The broad O-H stretching band shifts to 3422 cm^−1^, indicating hydrogen bonding interactions between hesperidin and the LDH hydroxyl groups. The C=O stretching vibration shifts from 1655 cm^−1^ to 1628 cm^−1^, suggesting electrostatic interactions between the carbonyl groups of hesperidin and the LDH layers. The C-O-C ether stretching bands shift from 1275 cm^−1^ and 1070 cm^−1^ to 1258 cm^−1^ and 1055 cm^−1^, respectively, indicating interactions between glycosidic bonds and the LDH matrix. The aromatic C=C vibrations remain present but show intensity variations, while the aromatic C-H stretching band shifts from 3100–3000 cm^−1^ to 3085 cm^−1^, and the aliphatic C-H stretching bands shift to 2965 cm^−1^, 2930 cm^−1^, and 2882 cm^−1^, suggesting van der Waals interactions between hesperidin and LDH. The C-H bending vibration at 823 cm^−1^ shifts to 815 cm^−1^, confirming structural modifications. The carbonate absorption band at 1360 cm^−1^ is reduced in intensity, indicating partial replacement of interlayer carbonate anions by hesperidin molecules, while the metal–oxygen vibrations below 1000 cm^−1^ remain unchanged, confirming the structural stability of LDH. These spectral variations confirm that hesperidin is successfully intercalated into the LDH layers through electrostatic interactions, hydrogen bonding, and van der Waals forces, leading to improved stability and controlled drug release potential.

### 3.2. HRTEM Analysis

HRTEM micrographs of MgAl-LDH and MgAl LDH-Hesp are displayed in Figure 2a,b, respectively. The intercalated counterpart, MgAl LDH-Hesp, displayed more elongated morphology with stacked layers’ agglomeration with a somewhat higher size range of 200–450 nm. The morphology of the LDH represented rhombohedral form in the size range between 100 and 200 nm. The intercalation of the Hesp into the interlayer gap caused a modest increase in the particle size of MgAl LDH–Hesp when compared to MgAl LDH.

### 3.3. Particle Size and Zeta Potential Analysis

**Figure 3:**
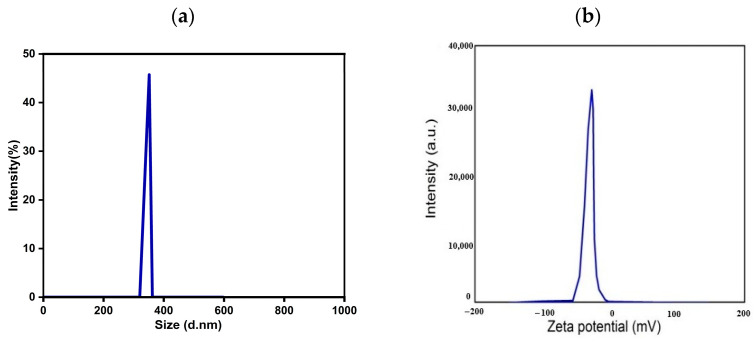
Measurements of (**a**) zeta sizer and (**b**) zeta potential MgAl LDH–hesperidin nanopowder.

Using dynamic light scattering (DLS) and a ZS90 Zeta sizer instrument (Malvern, UK), the hydrodynamic size of Hesp incorporated MgAl-LDH in the aqueous solution was determined. The results are shown in Figure 3a. The polydispersity index (PDI) of the aqueous solution of MgAl-LDH-Hesp was 0.513, indicating a particle size distribution of 350 nm with a mean size of 45 nm. Additionally, as seen in the HRTEM pictures (Figure 2), compared to the Z-average size, the zeta sizer measurement yielded a smaller value. The agglomeration of LDH in a solution could be the cause of the size discrepancy between HRTEM and DLS. With a refractive index of 1.330 and a zeta potential of −35 mv in water dispersion, MgAl-LDH-Hesp (Figure 3b) demonstrates reasonable stability.

### 3.4. Efficiency of Entrapment, Drug Loading, and In Vitro Drug Release Investigation

**Figure 4:**
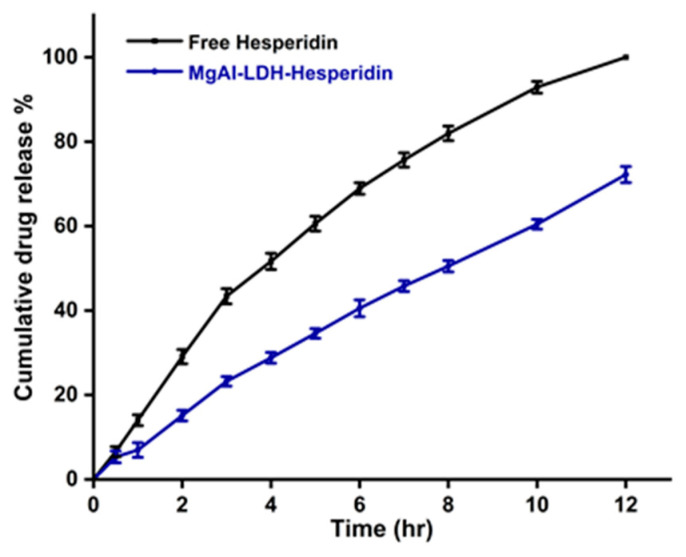
Profile of in vitro release of hesperidin from MgAl LDH–hesperidin in PBS at 37 °C. Error bars represent the standard deviation from three replicates. The difference in release was significant (*p* < 0.05) after 2 h.

The effectiveness of encapsulating and drug-loading of Hesp nanoparticles were 91.2 ± 2.4% and 12 ± 1.2%, respectively. Figure 4 shows that HPLC analysis was used to assess the Hesp release profile from the LDH at physiological pH (6.8) and a constant temperature of 37.5 °C in the phosphate buffer solution. Free hesperidin exhibits a rapid release, reaching approximately 100% within 12 h, which can be attributed to its high solubility and lack of a carrier system to modulate its dissolution. In contrast, Mg-Al LDH-loaded hesperidin shows a more controlled and sustained release, with cumulative release reaching around 72.21% at the same time point. This slower release is indicative of the strong interactions between hesperidin and the LDH matrix, likely involving electrostatic forces, hydrogen bonding, and interlayer entrapment, which hinder the immediate diffusion of the drug into the release medium. The release kinetics suggest that LDH acts as an effective drug delivery vehicle, reducing the initial burst release typically observed in free hesperidin and prolonging its availability over time. The sustained release profile of LDH-loaded hesperidin highlights the potential of the Mg-Al LDH system as a promising carrier for controlled drug release, which may enhance hesperidin’s bioavailability and therapeutic efficacy. These findings confirm that intercalation into LDH can significantly modify drug release characteristics, making it a suitable approach for sustained drug delivery applications.

### 3.5. Effects of Hesp and Hesp-NPs on APAP-Induced Liver Injury

**Figure 5:**
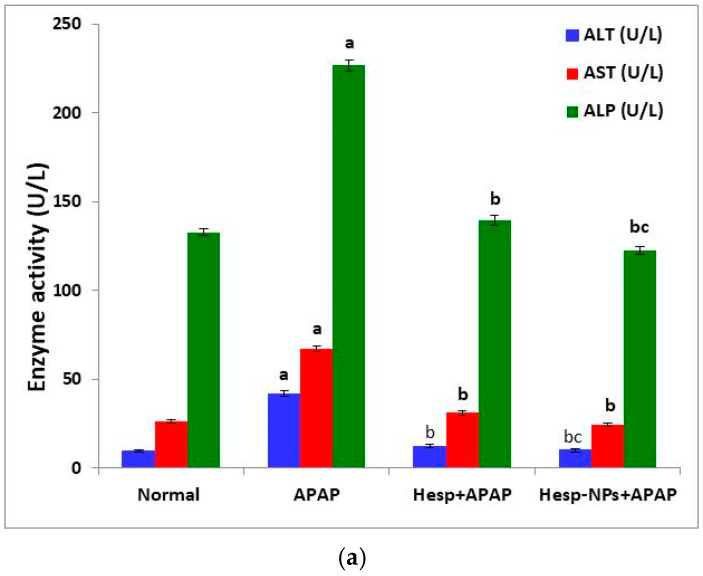

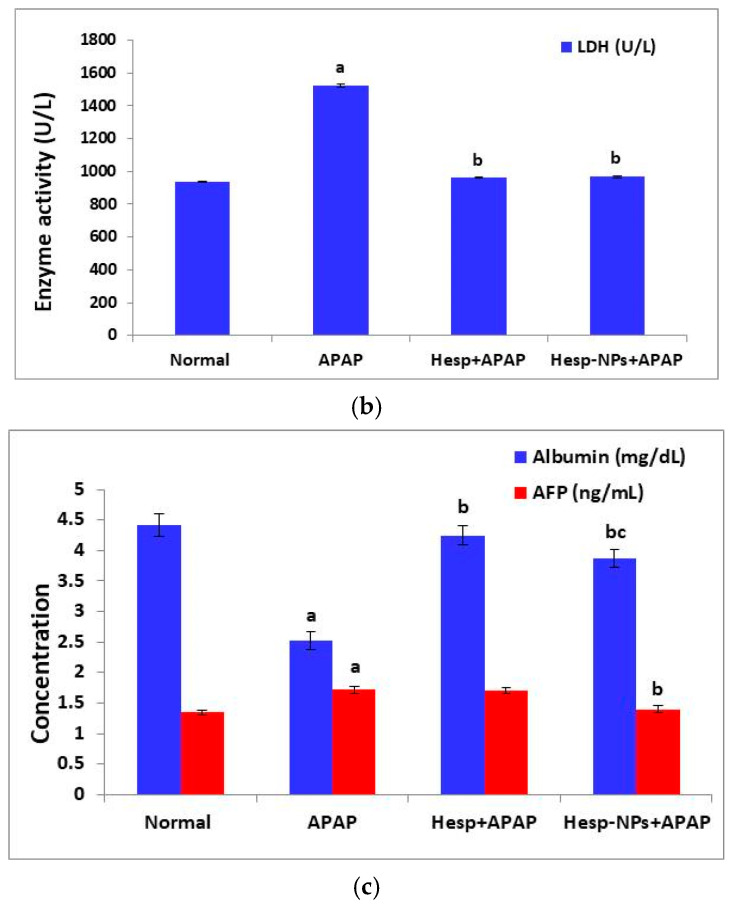
Effect of Hesp and Hesp-NPs on serum parameters measuring liver function, including ALT, AST, and ALP (**a**), LDH (**b**), albumin, and AFP (**c**) in rats given APAP. Data are presented as the mean ± SD. ^a,b^ and ^c^ indicate a significant change from control, APAP, and Hesp+APAP, respectively, at *p* < 0.05.

The APAP administration produced a significant increase (*p* < 0.05) in serum ALT, AST, ALP, and LDH activities. The treatment of APAP-supplied rats with Hesp and Hesp-NPs significantly (*p* < 0.05) prevented the increase in these enzyme activities; the treatment with Hesp-NPs seemed to be more potent (Figure 5).

The APAP administration also induced a significant increase in serum AFP levels (*p* < 0.05). The oral supplementation of Hesp-NPs successfully counteracted (*p* < 0.05) the APAP-induced increase in AFP level. On the other hand, the serum albumin level was significantly decreased (*p* < 0.05) by APAP oral administration and the treatment with Hesp and Hesp-NPs resulted in a significant increase (*p* < 0.05) in the lowered albumin level (Figure 5).

### 3.6. Effects of Hesp and Hesp-NPs on Liver GSH, MDA, and CAT Levels

**Figure 6:**
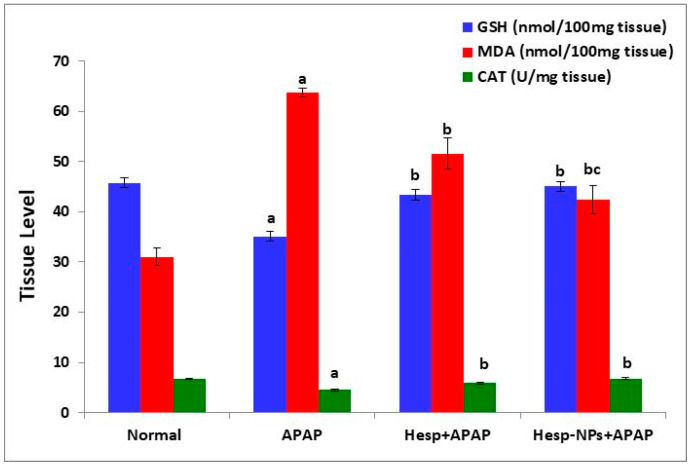
Effect of Hesp and Hesp-NPs on liver GSH, MDA, and CAT levels in rats given APAP. Data are presented as the mean ± SD. ^a,b^ and ^c^ indicate a significant change from control, APAP, and Hesp+APAP, respectively, at *p* < 0.05.

The APAP administration produced a significant decrease (*p* < 0.05) in liver GSH content and CAT activity. The treatment of APAP-supplemented rats with Hesp and Hesp-NPs significantly (*p* < 0.05) prevented this decrease (Figure 6). On the other hand, the MDA level was significantly increased (*p* < 0.05) by APAP oral administration and the treatment with Hesp and Hesp-NPs resulted in a significant decrease (*p* < 0.05) of the elevated MDA level (Figure 6).

### 3.7. Effects of Hesp and Hesp-NPs on Serum TNF-α and IL-4 Levels

**Figure 7:**
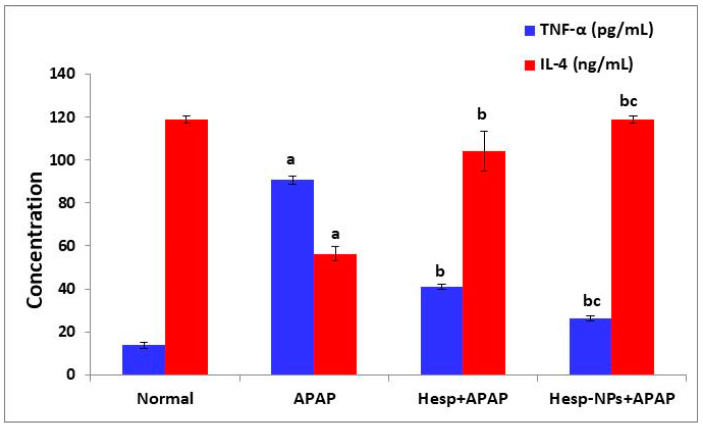
The effect of Hesp and Hesp-NPs on serum TNF-α and IL-4 levels in APAP-administered rats. Data are presented as the mean ± SD. ^a,b^ and ^c^ indicate a significant change from control, APAP, and Hesp+APAP, respectively, at *p* < 0.05. TNF-α: tumor necrosis factor-α.

The APAP-administered rats exhibited a significant increase (*p* < 0.05) in serum TNF-α level and a significant decrease (*p* < 0.05) in serum IL-4 level. The treatment of APAP-supplemented rats with Hesp and Hesp-NPs significantly (*p* < 0.05) prevented the TNF-α elevation and IL-4 depletion; the effect of Hesp-NPs was more potent than Hesp (Figure 7).

### 3.8. Effects of Hesp and Hesp-NPs on mRNA Expression of Various Genes

#### 3.8.1. Effects on mRNA Expression of Liver *CYP1A1* and UGTA6

**Figure 8:**
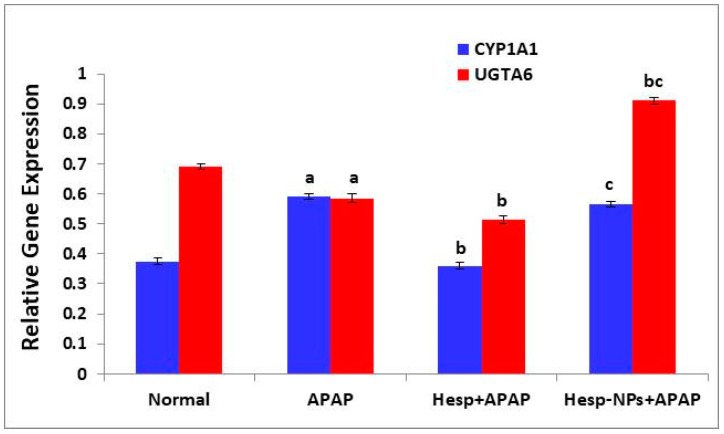
Effect of Hesp and Hesp-NPs on mRNA expression of *CYP1A1* and UGTA6 in liver of APAP-administered rats. Data are presented as the mean ± SD. ^a,b^ and ^c^ indicate a significant change from control, APAP, and Hesp+APAP, respectively, at *p* < 0.05.

The APAP administration produced a significant increase (*p* < 0.05) in the expression of liver *CYP1A1* and a significant decrease (*p* < 0.05) in the expression of liver UGTA6. The treatment of APAP-supplemented rats with Hesp and Hesp-NPs resulted in a significant decrease (*p* < 0.05) in the elevated *CYP1A1* expression (Figure 8). The effect of Hesp was more potent than Hesp-NPs. The UGTA6 expression was significantly decreased (*p* < 0.05) by the treatment with Hesp, while it was significantly increased (*p* < 0.05) by Hesp-NPs (Figure 8).

#### 3.8.2. Effects on mRNA Expression of Liver *HO-1*, *NQO1*, *NQO2*, and *KEAP1*

**Figure 9:**
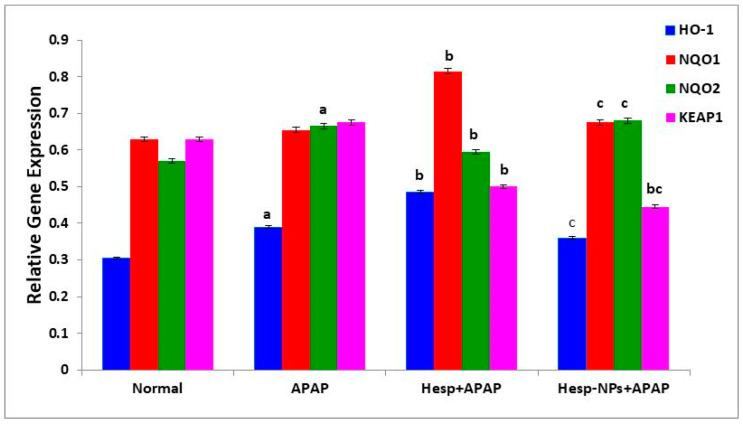
Effect of Hesp and Hesp-NPs on mRNA expression of *HO-1*, *NQO1*, *NQO2*, and *KEAP1* in liver of APAP-administered rats. Data are presented as the mean ± SD. ^a,b^ and ^c^ indicate a significant change from control, APAP, and Hesp+APAP, respectively, at *p* < 0.05 using ANOVA, followed by LSD test.

The APAP administration produced an increase in the mRNA expression of *HO-1*, *NQO1*, *NQO2*, and *KEAP1* in the liver; the effect was significant (*p* < 0.05) on *HO-1* and *NQO2*. The APAP-supplemented rats treated with Hesp exhibited a significant increase in *HO-1* and NQO1 expression when compared with APAP-administered control, while Hesp-NPs resulted in a significant decrease (*p* < 0.05) in comparison to the Hesp+APAP group. The NQO2 expression was significantly decreased (*p* < 0.05) by the treatment with Hesp. Keap1 expression was significantly (*p* < 0.05) down-regulated by Hesp and Hesp-NPs, which was more effective (Figure 9).

#### 3.8.3. Effects on mRNA Expression of Liver mRNA Expression of *TGF-β* and *IL-10*

**Figure 10:**
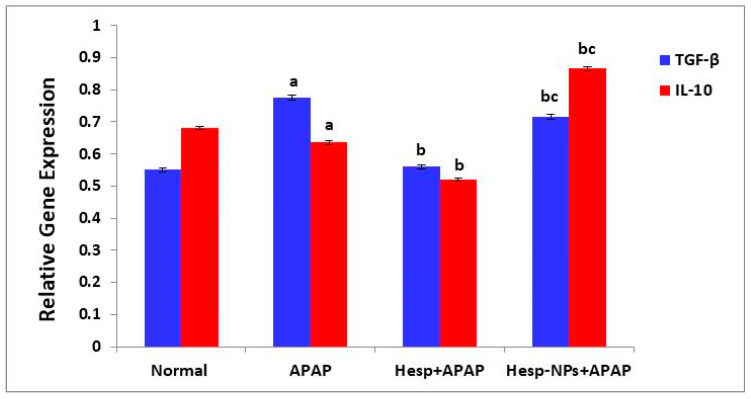
Effect of Hesp and Hesp-NPs on mRNA expression of *TGF-β* and *IL-10*. Data are presented as the mean ± SD. ^a,b^ and ^c^ indicate a significant change from control, APAP, and Hesp+APAP, respectively, at *p* < 0.05 using ANOVA followed by LSD test.

The APAP administration produced a significant increase (*p* < 0.05) in liver mRNA expression of *TGF-β*. The treatment of APAP-supplemented rats with Hesp and Hesp-NPs resulted in a significant (*p* < 0.05) decrease; Hesp was more effective (Figure 10).

Liver *IL-10* expression was significantly decreased by APAP administration. The treatment of APAP-supplemented rats with Hesp-NPs resulted in a significant (*p* < 0.05) increase, while Hesp induced a significant decrease in comparison to APAP control (Figure 10).

#### 3.8.4. Effects on mRNA Expression of Markers Related to Apoptosis (*P53* and *BAX*) in the Liver

**Figure 11:**
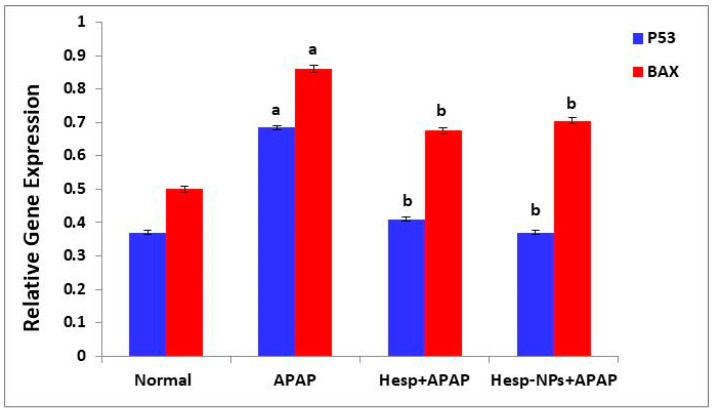
Effect of Hesp and Hesp-NPs on mRNA expression of markers related to apoptosis (*P53* and *BAX*) in liver of APAP-treated rats. Data are presented as the mean ± SD. ^a,b^ indicate a significant change from control, APAP, and Hesp+APAP, respectively, at *p* < 0.05.

The APAP administration produced a significant increase (*p* < 0.05) in the mRNA expression of *P53* and BAX in the liver. The treatment of APAP-supplemented rats with Hesp and Hesp-NPs resulted in a significant (*p* < 0.05) decrease. Hesp-NPs were more effective in decreasing the elevated *P53* expression (Figure 11).

### 3.9. Effects on Liver Histological Changes

**Figure 12:**
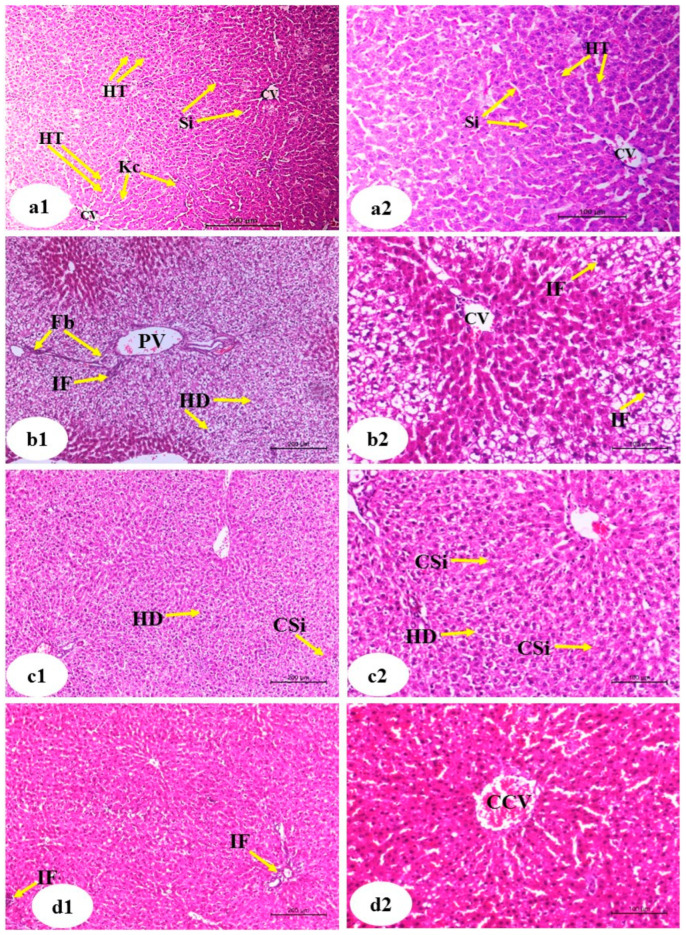
Photomicrographs of liver sections of normal ((**a1**), low magnification ×100; (**a2**), high magnification of a1 ×200), APAP ((**b1**), low magnification ×100; (**b2**), high magnification ×200), Hesp+APAP ((**c1**), low magnification ×100; (**c2**), high magnification ×200) and Hesp-NPs+APAP ((**d1**), low magnification ×100; (**d2**), high magnification ×200) groups. CV, central vein; HT, hepatic trabeculae; Si, sinusoids; HP, hepatic portal vein; HD, hydropic degeneration; CSi, congested sinusoids; Kc, Kupffer cells; Fb, fibroblast; IF, inflammatory infiltration; CCV, congested central vein.

Figure 12 shows histological changes in the liver. Liver sections from the normal control animals revealed typical hepatic cells arranged in hepatic strands or trabeculae radiating towards the central vein and enclosing sinusoids in between (Figure 12(a1,a2)). Sections from the APAP-treated animals revealed severe bridging hydropic degeneration, fibroblast proliferation, inflammation, and sinusoidal expansion (Figure 12(b1,b2)). Simultaneous treatment with Hesp and APAP (Figure 12(c1,c2)) showed normal hepatocyte morphology with mild sinusoidal congestion and mild hydropic degeneration. Rats in the group treated with APAP plus Hesp-NPs (Figure 12(d1,d2)) showed normal hepatic lobular architecture with mild inflammation and central vein congestion. Furthermore, liver injury demonstrated in rats treated with Hesp and Hesp-NPs had fewer inflammatory reactions, leading to a lower degree of necrosis in comparison to the paracetamol group.

Table 2 shows the histopathological scores of hydropic and vacuolar degeneration, inflammation, fibroblast proliferation, and vascular and sinusoidal congestion. The normal liver sections exhibited a 0 score for all these histological lesions. APAP groups treated with Hesp and Hesp-NPs depicted lower scores of histological lesions of all assessed lesions than APAP control; Hesp-NPs were more potent than Hesp.

## 4. Discussion

Because the liver is the primary organ involved in the elimination, metabolism, and excretion of several exogenously and endogenously supplied medicines and harmful chemicals, it is more vulnerable to injury and damage. Injury to the liver can be caused by over-the-counter (OTC) drugs as well as by prescription medicines [41,42]. The need to develop a hepatoprotective agent of natural sources using nanotechnology was our main interest in the current study. As mentioned, a nanoformula consisting of Mg-Al-LDH loaded with Hesp nanoparticles was prepared to be investigated as a hepatoprotective agent versus a toxic APAP.

A common target for the hepatotoxicity of several medications is the mitochondria [43]. When this essential cell organelle deteriorates, energy metabolism is hampered and intracellular oxidant stress with increased ROS production occurs [44]. Apart from mitochondria, oxidative stress and cell damage are also induced by the stimulation of cytochrome P450 isoenzymes [45]. The expression of liver *CYP1A1*, which is a cytochrome P450 Family 1 Subfamily a Member 1, increased significantly as a result of APAP administration in the current study. The expression of liver UGTA6, which augments the antioxidant, glucuronidation, and detoxification response, was significantly down-regulated in APAP-administered rats. The stimulation of *CYP1A1* and suppression of *UGTA6* expressions were associated with an increase in oxidative stress and ROS production. This was evidenced in the present study by an increase in lipid peroxidation and a decrease in liver GSH content and CAT activity. The treatments with Hesp and Hesp-NPs successfully prevented these deteriorations.

The liver metabolizes APAP nearly entirely. The remainder undergoes phases of sulphation, glucuronidation, oxidation, and thiolation. Since APAP overdoses have consequences, they have been known to cause fulminant hepatic failure, and both long- and short-term use have been linked to increases in the leakage of liver transaminases into blood, indicating acute liver injury [46]. The hepatotoxicity of paracetamol overdose was confirmed by the significantly higher levels of the enzymes AST, ALT, LDH, and ALP in the serum of the APAP group compared to the control group. These findings corroborated those of Iqbal Dar et al., who indicated that increased levels of biochemical markers such as AST, ALP, ALT, and serum bilirubin were indicative of liver injury brought on by excessive paracetamol use [4]. This is because hepatic cells have several different metabolic processes and are home to a wide range of enzymes. Higher concentrations of ALT and AST were discovered in the cytoplasm of hepatocytes, with AST being concentrated in the mitochondria, but the mitochondrial ALT isoform is not found. When there is liver damage, the hepatocytes’ transport function is compromised, which leads to plasma membrane leakage and the subsequent release of these enzymes, which raises their levels in the blood. Hesp and Hesp-NPs administration improved the measured markers, preventing liver damage caused by paracetamol. On the other hand, albumin was significantly decreased, confirming the hepatotoxicity of the APAP overdose. The treatments of APAP-administered rats with Hesp and Hesp-NPs successfully restored the decreased albumin level. AFP serves as a prognostic and diagnostic marker. The current investigation’s findings revealed that the group treated with paracetamol had higher levels of AFP than the control. After hepatic necrosis, an increase in AFP is thought to be a sign of hepatic regeneration [47]. Elevated AFP levels have been linked to liver damage, and in individuals with chronic hepatitis B, they rose in tandem with pathological levels of inflammation and fibrosis. In the current study, serum AFP level was significantly elevated in the APAP-administered group and in the Hesp+APAP-administered rats. However, it was not significantly affected in Hesp-NPs+APAP-treated rats when compared with normal control. This led us to suggest successfully normalizing the AFP production and, thereby, their levels.

Given that the endogenous antioxidant mechanism is frequently disrupted, resulting in significant tissue damage, it is widely recognized that oxidative stress is strongly linked to the etiology of hepatotoxicity [46,48]. The APAP group had greater MDA levels than the control group. The pre-administration of Hesp and Hesp-NPs significantly reduced MDA levels in comparison to the APAP group. APAP administration resulted in a considerable reduction in the liver’s GSH content and a marked decrease in CAT in comparison to the control. In contrast to the APAP group, pretreatment with Hesp and Hesp-NPs-treated cells enhanced the GSH and CAT content.

It is worth mentioning that APAP metabolism produces the reactive intermediate NAPQI forming mitochondrial protein adducts, which induce oxidative stress within the organelle and set off signaling cascades that ultimately lead to programmed necrosis [49]. The resultant toxic NAPQI byproduct is converted into nontoxic metabolites by GSH; GSH is a major antioxidant system and a crucial component of host defense [50,51]. It is responsible for scavenging reactive free radicals produced through the metabolism process within the liver to prevent cell injury, and this is a possible explanation for why GSH decreased in the APAP group [52]. Furthermore, the presented results were in agreement with others’ findings, which showed the function of mitochondria in oxidative stress caused by APAP. In APAP overdose, the oxidation of APAP to the corresponding phenoxyl free radical and NAPQI by peroxidases results in the increased production of ROS and free radicals while GSH content decreases, NAPQI accumulates as a result of the toxic dose of APAP, depleting GSH [52]. NAPQI subsequently forms NAPQI-protein adducts by covalently binding to the cysteinyl sulfhydryl groups of cellular proteins [1]. Increased levels of free radicals cause protein oxidation, lipid peroxidation, and enzymatic inactivation; that is why the MDA (as a measure of lipid peroxidation) increased while the CAT enzyme decreased in the APAP group.

One of the primary tissue antioxidant defense mechanisms against free radicals is the enzyme CAT. Oxidative stress and tissue damage are caused by insufficient CAT, which can be attributed to either down-regulation of gene expression or consumption [53,54]. Lipid peroxidation of cell membrane lipids is induced by any form of oxidative stress to a cell. The results of this study, which show decreased CAT activity and an increase in lipid peroxidation, are in line with many animal studies where hepatocellular damage caused by APAP has been reported [55,56,57]. The peroxidative products are toxic and inflict extensive damage to macromolecules. Together with our current findings, these data suggest that the hepatoprotective effect of Hesp and Hesp-NPs treatment is most likely achieved by functioning as antioxidants and preventing lipid peroxidation [55].

This study confirmed that Hesp exhibited antioxidant activity. To illustrate a convenient mechanism of Hesp’s antioxidant activity, Hesp’s structure–activity relationship and flavonoids’ antioxidant qualities are related to their capacity to scavenge free radicals, chelate metals, and limit oxidase activity. It has not been clearly established, though, how Hesp’s structural activity relates to their antioxidative action. The ability of flavonoids to transfer an electron or a hydrogen atom, as well as the potential for interactions with other antioxidants, are generally referred to as their antioxidant activities [10].

Numerous efforts have been documented in the literature to elucidate the links between the structure and action of certain natural antioxidant molecules. According to reports, phenolic compounds’ antioxidant activity can come from their capacity to donate hydrogen, which neutralizes free radicals and stops oxidation processes or radical chain reactions [57]. Additionally, it is well-recognized that structural modifications to polyphenolic compounds, such as those involving side-chain and aromatic ring replacements, have a direct impact on their antioxidant activity. The quantity and location of phenolic hydrogens inside polyphenolic compounds are thought to affect their capacity to scavenge free radicals [58]. Additionally, it is suggested that the increased amount of hydroxyl groups in the flavonoid nucleus correlates with higher antioxidant activity [59]. It is noteworthy that flavonoids have largely different biological effects with very few structural variations despite their structural similarities. The flavonoids act as potent antioxidants, anti-inflammatories, and antiproliferatives [60,61]. The most well-known reactive chemical species among oxygen radicals is the hydroxyl radical. Biomolecules like DNA, all proteins, nucleic acids, and nearly every other biological molecule are susceptible to some degree of oxidative damage from the hydroxyl radical. Nevertheless, nothing is known about Hesp’s antioxidative properties. Based on the chemical structure of Hesp (Scheme 1), agents that modify enzymes. The most well-known reactive chemical species among oxygen radicals is the hydroxyl radical. Biomolecules like DNA, all proteins, nucleic acids, and nearly every other biological molecule are susceptible to some degree of oxidative damage from the hydroxyl radical. It has been reported that Hesp has antioxidant, anti-inflammatory, and anticancer activities [62,63].

The development of immunological responses is intimately associated with the incidence of paracetamol-induced liver damage. As illustrated by a considerable rise in the hepatic inflammatory markers comparable to those in normal rats, APAP administration induced significant damage. During acute inflammation, monocytes and macrophages release a cytokine called TNF-α, which is a crucial pro-inflammatory cytokine. TNF-α controls blood coagulation and increases oxidative stress in inflammatory sites [64]. Elevated TNF-α levels due to APAP oral administration were dramatically lowered by oral treatment with Hesp and Hesp-NPs. This indicates that Hesp and Hesp-NPs function as TNF-α inhibitor medications to limit inflammation. When TNF-α inhibitors are injected into the bloodstream, the immune system responds by preventing inflammation. These results are in accordance with Emam and Madboly [65], who revealed that hesperidin prevented APAP-induced elevation in serum TNF-α levels. The increase in the anti-inflammatory cytokine IL-4 serum level in the APAP-administered rats due to treatments with Hesp and Hesp-NPs provides another evidence for their anti-inflammatory roles. In addition, the treatment of APAP-administered rats with Hesp-NPs significantly enhanced the expression of liver *IL-10*, which is another Th2 (T helper 2) anti-inflammatory cytokine. In concurrence with this study, the administration of Hesp led to an increase in the levels of serum IL-4 (as an anti-inflammatory cytokine) and a decrease in TNF-α (as a pro-inflammatory and inflammatory cytokine) in many animal models of diseases [66,67,68,69]. The effect of Hesp-NPs was significantly more effective on serum TNF-α and IL-4 levels than free Hesp. The prolonged and sustained release of Hesp from Mg-Al-LDH nanocarriers may be responsible for Hesp-NPs potent anti-inflammatory properties. Enhanced transport to, or uptake by, target cells may be another reason for the nanocomposite’s superior impact in addition to sustained release [70,71,72]. Since inflammation plays a significant role in triggering APAP-hepatotoxicity [73] and Hesp-NPs have a stronger anti-inflammatory impact than Hesp, this could account for the nanocomposite’s superior ability to protect against APAP-hepatotoxicity in the present study.

Regarding the histological changes, sections from the APAP-treated animals revealed severe bridging hydropic degeneration, inflammation, and sinusoidal expansion. Simultaneous treatment with Hesp and APAP showed normal hepatocyte morphology with mild sinusoidal congestion and mild hydropic degeneration. Rats of the group treated with APAP plus Hesp-NPs showed normal hepatic lobular architecture with the absence of mild inflammation and central vein congestion. The histological alterations in the liver, such as inflammation in the hepatic lobules and degeneration of centrilobular hepatocytes, are due to APAP toxicity. These data agreed with Nathiya et al., who revealed that, without a doubt, Hesp lessens liver damage, degeneration, and liver cell necrosis [42]. Furthermore, liver injury demonstrated in rats treated with Hesp and Hesp-NPs had fewer inflammatory reactions, leading to a lower degree of necrosis in comparison to the paracetamol group. Also, Chen et al. showed that Hesp suppressed congestion and vacuolar degeneration in the liver of nickel-intoxicated mice [74]. Çetin et al. found that Hesp produced amelioration of histological lesions, including hepatocellular necrosis, hemorrhage, mononuclear cell infiltration, vascular congestion, eosinophilic and pyknotic nuclei hepatocytes, as well as vacuolated hepatocytes [16]. Furthermore, Abdelaziz et al. revealed that Hesp remarkably attenuated inflammatory response in methotrexate-induced liver injury [75]. They attributed the hepatoprotective nature of Hesp against methotrexate hepatotoxicity to its ability to suppress the pro-inflammatory and apoptotic mediators and to enhance the antioxidant defense system.

Apoptosis is a process of controlled cell death that is essential to many biological processes, such as multicellular organisms’ cell development, proliferation, and differentiation, as it aids in the removal of undesirable, damaged, or contaminated cells [76]. The apoptosis process is regulated by certain parameters, and the standing research investigated the apoptotic parameters at the level of gene expression. We measured *P53* and *BAX* expressions as checkpoints for apoptosis.

Both Bcl2 homologous antagonist/killer (Bak) and Bax proteins are recognized as crucial entry points for cell death. Bax, the protein expressed by the *BAX* gene, is believed to be the primary cell executioner and is crucial for cell death in a variety of situations, including embryonic hypoxia, apoptosis, oxidative stress, ischemia, potassium deprivation, and *P53* overexpression. Bax and proapoptotic executioner proteins called Bak proteins cause apoptosis by creating mitochondrial permeability transition holes that let cytochrome c release [77]. The present study’s findings demonstrate an increase in *BAX* expression in the APAP-induced apoptosis. On the other hand, both Hesp and Hesp-NPs down-regulated the *BAX* mRNA expression with a significant pattern (*p* < 0.05). Thus, these findings provide evidence for the anti-apoptotic effects of Hesp and Hesp-NPs. Our result agrees with Tabeshpour et al. study, which manifested that the expression of the proapoptotic gene *BAX* increased. Hesp (200 mg/kg, p.o., for 6 days) mitigated the paraquat hepatotoxicity by its anti-apoptotic, anti-inflammatory, and antioxidant properties [57]. The proapoptotic gene *BAX*, in conjunction with the suppression of *BCL2*, is the gene known to protect cells against death [78].

The tumor suppressor gene *P53* encoded the p53 protein, also referred to as the tumor suppressor protein [79]. The tumor suppressor protein p53 is essential for controlling the cell cycle, apoptosis, senescence, and metabolism in response to many triggers, including DNA damage and cellular stress. As p53 facilitates cell survival and genetic damage repair once it is activated, it should come as no surprise that oxidative stress in APAP hepatotoxicity also activates p53. By preventing the JNK pathway from being activated, activated p53 provides protection during the injury phase of APAP hepatotoxicity but also slows down the healing process during the regeneration phase [80]. p53 expression was significantly up-regulated in APAP-administered rats, manifesting the apoptotic effects of over high dose of APAP. Conversely, when compared to the APAP-injected control, the Hesp and Hesp-NPs therapies resulted in a significant down-regulation (*p* < 0.05) in P53 mRNA expression; Hesp-NPs treatment was more effective. These results are in accordance with many publications [73,75,80]. The sustained and prolonged release of Hesp from MgAl-LDH nanoparticles during the time interval between every two consecutive doses may have an important role in producing a more potent anti-apoptotic effect of Hesp nanocomposite than its free form, as indicated by the in vitro investigation of the present study. The enhanced transport to, or uptake by, liver cells may be another reason [63,64,65].

As both the proteins expressed by the genes BAX and P53 are mediators in the intrinsic pathway of apoptosis and are proapoptotic proteins, the down-regulating effects of Hesp and Hesp-NPs provide evidence for the anti-apoptotic effects of these treatments. The anti-apoptotic effects of Hesp and Hesp-NPs have an important role in mediating the hepatoprotective effects of these agents.

The Kelch-like ECH-associated protein 1 (Keap1)/nuclear factor erythroid 2-related factor 2 (Nrf2)/oxidative responsive elements (ARE) (Keap1/Nrf2/ARE) pathway is a major defense mechanism versus oxidative stress and is crucial to the etiology and development of numerous disorders [81]. The transcription factor Nrf2 is a key modulator of antioxidant cellular responses and a phytochemical target that offers defense against disorders linked to oxidative stress. KEAP1 is the main negative regulator of the *NRF2* gene and mediates ubiquitylation and degradation of Nrf2 [82]. When Nrf2 is not under stress, Keap1, a component of the E3 ligase, attaches to the protein in the cytoplasm and facilitates its ubiquitination and proteasome-dependent destruction. Nrf2 separates from Keap1 in reaction to stress, stabilizes, and then builds up in the nucleus, activating the specific ARE found in gene promoters. The expression of several antioxidant and detoxification enzyme genes, such as *NQO1* and *HO-1*, is induced by Nrf2, and it also triggers a variety of cell defense mechanisms, which improve the cells’ overall ability to detoxify and remove toxins [83]. The current study’s findings showed that APAP elevated the expression of *KEAP1*, but Hesp and Hesp-NPs down-regulated *KEAP1* expression, which in turn caused Nrf2 to become activated. The activation of Nrf2, in turn, enhanced the expression of antioxidant enzymes and suppressed oxidative stress and inflammation, leading to improvement in liver function and histological integrity. According to Jiang et al., rats given APAP had a considerably higher cytoplasmic expression of *KEAP1*, which was subsequently decreased by Sanghuangporus sanghuang [84].

Enzymes known as cytochrome P450 (CYP450) are essential for both medication metabolism and the detoxification of foreign substances. The reason CYP450 enzymes get their name is that they are attached to cell membranes (cyto) and have heme pigments (chrome and P) that, when exposed to carbon monoxide, absorb light with a wavelength of 450 nm [85]. The heme-containing monooxygenase CYP450, which is mostly found in the liver, is thought to have a catalytic and regulatory role in APAP metabolism and is a valuable target for therapeutic intervention in metabolic regulation [86]. One of the key members of the CYP1A family and one of the several isoforms of CYP450 is CYP1A1 enzyme, which is in charge of metabolically activating procarcinogens (heterocyclic amines, aromatic amines, and polycyclic aromatic hydrocarbons) into reactive metabolites. Additionally, it is involved in the metabolism of estrogens and other steroidal hormones. The current study’s findings showed that Hesp and Hesp-NPs reduced the hepatotoxicity caused by chemical toxicants, such as APAP, by down-regulating the expression of the *CYP1A1* enzyme, as shown in Figure 8. These results agreed with a previous study that demonstrated that HSP reduced B[a]P-induced lung cancer by mitigating the expression of *CYP1A1* [87].

Phase I enzymes activate xenobiotic chemicals (drugs, environmental contaminants), phase II enzymes conjugate them, and phase III transporters remove them from the body through urine or bile. Phase II conjugating enzymes encompass a variety of enzyme superfamilies, including uridine diphosphate-glucuronosyltransferase (UGT), sulfotransferase, and glutathione S-transferase (GST). Phase I enzymes are mostly members of the cytochrome P450 (CYP) superfamily. When CYP metabolically activates some xenobiotics, unhealthy intermediates are produced. The metabolism depends on the conjugation reaction [88]. Since UGT conjugates and excretes in urine or bile (55% of the 200 most commonly prescribed medications), the UGT superfamily is the most significant group of phase II conjugating enzymes in xenobiotic metabolism [89]. In addition, bilirubin, steroid hormones, thyroid hormones, bile acids, and fat-soluble vitamins are among the numerous endogenous substances that UGT conjugates. The conjugation of glucuronic acid’s glycosyl group to numerous lipophilic endogenous and foreign substances is catalyzed by UGT. A comparison of amino acid sequences has led to the division of the UGT superfamily into two major groups, known as UGT1 and UGT2 [88]. The metabolism of drugs is aided by the UGT1A and UGT2B subfamily enzymes. Either the bilirubin group (UGT1A1 through 1A5) or the phenol group (UGT1A6 through 1A10) can be distinguished between the rodent and human *UGT1* genes according to similarities in sequence and substrate selectivity. The current study investigated the *UGT1A6* in UGT xenobiotic metabolism. The results revealed a significantly high expression of *UGT1A6* in Hesp-NPs only compared to APAP, while in the Hesp group, the expression of UGT1A6 was low (Figure 8). These data agreed with Stingl et al., which showed that the glucuronidation of paracetamol is mediated by UGT1A1, 1A9, 1A6, and 2B15. The primary enzyme involved in its metabolism is UGT1A6, and the allele UGT1A6 increases the activity of the enzyme [89]. Asians with the 1A6 allele have been shown to have nearly double paracetamol clearance. These findings suggest that the *UGT1A6* polymorphism may have a clinically significant impact.

The two members of the mammalian quinone oxidoreductases family, NQO1 and NQO2, are in charge of reducing quinones or quinone-like compounds. Quinones are an organic compound class that is formed from aromatic compounds. They are a part of the quinoid family, which also includes quinone methides and quinone imines [90]. NQO1 and NQO2 are two essential enzymes for detoxification that shield cells from oxidative injury [90,91]. Both enzymes can be triggered by oxidative stress, APAP, or semiquinone radicals, including NAPQIs. When other detoxification routes are depleted, the activity of both antioxidant enzymes, NQO1 and NQO2, becomes very crucial. As a result, this article provides an overview of how NQO1 and NQO2 interact with various pharmacological agents, endogenous biochemicals, and environmental contaminants. This information may be helpful in the development of treatments that lessen the risk of quinone-induced oxidative injury and minimize adverse drug reactions [91]. Through its ability to bind with other proteins, such as p53, NQO1 can prevent 20S proteasomal degradation of other proteins. This shows that maintaining the stability of vital proteins like p53 and other transcription factors is one significant function of NQO1 when it is produced under stress [92]. In this regard, both *NQO1* and *P53* expressions were elevated in the liver by an overdose of APAP in the current study. Miettinen and Björklund demonstrated that APAP and numerous APAP-like substances have a unique off-target in the cytosolic protein NQO2 [93]. In cultured cells, APAP-induced superoxide generation is mediated by *NQO2*, a protein that is widely expressed in the liver and kidneys of humans [84]. In accordance, the current study evidenced that *NQO2* expressions were significantly increased in APAP-administered rats in association with an increase in oxidative stress. On the other hand, the treatment with Hesp induced a significant increase in *NQO1* expression compared to APAP, while the treatment with Hesp-NPs did not. In contrast, liver expression of the NQO2 enzyme exhibited a significant decrease in the APAP group treated with Hesp only.

HO-1 is responsible for the breakdown of toxic and oxidative-free heme into free iron, carbon monoxide (CO), and biliverdin, which are then transformed into bilirubin. HO-1 contributes significantly to the reduction of inflammation in addition to its antioxidant role. Consequently, it is currently thought that pharmacologically up-regulating *HO-1* gene expression offers a unique targeted treatment [94]. The results in the current study revealed a high expression of *HO-1* gene in the Hesp group compared to APAP, while in the Hesp-NP group, the expression of *HO-1* gene was lower compared to APAP. In consistence with these results, Chen et al. found that Hesp has the capacity to stimulate *HO-1* expression in L02 cells in a dose-dependent manner [95].

Multiple peptides in the TGF-β family control various aspects of cell life, including migration, adhesion, differentiation, and proliferation. In addition to promoting the formation of cells, profibroblats, and fibroblasts, TGF-β level was found to promote the deposition of the extracellular matrix (ECM) components and was considered a marker of fibrosis [96]. In the liver, Kupffer and endothelial cells synthesize a substantial amount of TGF-β [97]. In association with the fibroblastic proliferation in the liver, *TGF-β* mRNA gene expression significantly increased in the group supplied with APAP compared to the normal control group. The current results also revealed a significant down-regulation of *TGF-β* expression due to Hesp and Hesp-NPs treatments compared to APAP. In accordance with the present study, Pérez-Vargas et al. stated that the decrease in *TGF-β* expression has a role in preventing liver fibrosis by hesperidin [98].

Given that elevated IL-10 levels may be a result of a defensive mechanism to reduce harmful inflammation [99], it is mysterious how APAP affects the inflammatory response because, in our trial, it considerably lowered the amount of IL-10. Interventions to lower the level of these biomarkers did not improve patient outcomes, although there is a possibility that pyrogenic cytokines may play a role in the febrile response and physiologic abnormalities brought on by specific infections. This finding is consistent with earlier findings and our own investigation [100]. In the APAP group, there was a significant drop in *IL-10* mRNA expression, but this difference did not persist in the group receiving no therapy. As seen in Figure 10, the results showed that *IL-10* mRNA expression was low in the Hesp group and significantly higher in Hesp-NPs only when compared to APAP. In our opinion, this effect may be due to the sustained release of Hesp from the nanocomposite during the time interval between each two consecutive doses. The enhanced penetration of the nanocomposite into liver cells may be another reason.

## 5. Conclusions

Hesp-MgAl-LDH protected the liver against APAP-caused oxidative stress, lipid peroxidation, inflammation, and apoptosis. We discovered that rats given APAP in conjunction with Hesp-NPs avoid hepatotoxicity. Future research ought to look into the possibility that liver-specific Hesp therapy could be a useful tactic for lowering hepatotoxicity at the molecular level. Hesp nanostructure, prolonged release, and LDH-trapping efficiency could all contribute to Hesp-NPs’ increased biological activity. According to the current study, the primary cause of Hesp and Hesp-NPs’ strong protective action against APAP-caused hepatotoxicity may be due to their antioxidant, anti-inflammatory and anti-apoptotic qualities. Thus, Hesp-NPs provide a potentially effective strategy for managing the hepatotoxicity of OTC drugs like APAP. However, further clinical studies are required to assess the efficacy of Hesp-NPs against drug-induced hepatotoxicity as well as the safety in human beings.

## Figures and Tables

**Table 1:** Primers’ sequences of detected genes.

Genes	Forward Sequence (5′-3′)	Reserve Sequence (5′-3′)	Ref.
*β-ACTIN*	TCACCCTGAAGTACCCCATGGAG	TTGGCCTTGGGGTTCAGGGGG	[32]
*BAX*	GACACCTGAGCTGACCTTGG	GAGGAAGTCCAGTGTCCAGC	[33]
*P53*	CAGCGTGATGATGGTAAGGA	GCGTTGCTCTGATGGTGA	[34]
*KEAP-1*	GGACGGCAACACTGATTC	TCGTCTCGATCTGGCTCATA	[35]
*CYP1A1*	GTGGCCTGTATTTTGCTTATG	AGCTCAGGTACGTTTTTCCTA	[35]
*NQO1*	GGAGACTGTCTGGGAGGAGT	GCTTTGATCTGGTTGTCGGC	[36]
*NQO2*	TCCAGGAGGCAGAGACTGTT	AGAGGGCACCAGTAACATCG	[36]
*UGTA1*	ACTCAAAGTATGAGATCCTTGC	TCAAATTCCTGAGACAGGTTC	[35]
*TGF-β*	TGAGTGGCTGTCTTTTGACG	TGGGACTGATCCCATTGATT	[37]
*HO-1*	CAGAGTTTCTTCGCCAGAGG	TGAGTGTGAGGACCCATCG	[38]
*IL-10*	GCAGGACTTTAAGGGTTACTTGG	GGGGAGAAATCGATGACAGC	[39]

*BCL2*, B-cell lymphoma 2; *BAX*, *BCL2* Associated X; *P53*, *tumor protein 53*; *KEAP-1*, Kelch-like ECH-associated protein-1; *CYP1A1*, cytochrome P450 1A1; *NQO1*, NAD(P)H quinone oxireductase-1; *NQO2*, NAD(P)H quinone oxireductase-2; *UGTA1*, uridine diphosphate glucuronosyltransferase 1A1; *TGF-β*, transforming growth factor-β; *HO-1*, Heme oxygenase 1; *IL-10*, interleukin 10.

**Table 2:** Histopathological scores of liver lesions in normal, APAP, Hesp+APAP, and Hesp-NPs+APAP groups.

Histopathological Changes	Score	Normal	APAP	Hesp + APAP	Hesp-NPs + APAP
Hydropic and vacuolar degeneration	0	6 (100%)	-	-	1 (16.7%)
	I	-	-	2 (33.3%)	4 (66.7%)
	II	-	-	4 (66.7%)	1 (16.7%)
	III	-	6 (100%)	-	-
Inflammation (inflammatory cell infiltration)	0	6 (100%)	-	1 (16.7%)	3 (50.0%)
	I	-	1 (16.7%)	3 (50.0%)	2 (33.3%)
	II	-	3 (50.0%)	1 (16.7%)	1 (16.7%)
	III		2 (33.3%)	1 (16.7%)	-
Fibroblast proliferation	0	6 (100%)	-	1 (16.7%)	4 (66.7%)
	I	-	3 (50.0%)	5 (83.3%)	2 (33.3%)
	II	-	2 (33.3%)	-	-
	III		1 (16.7%)	-	
Congested blood vessels and sinusoids	0	6 (100%)	-	3 (50.0%)	4 (66.7%)
	I	-	2 (33.3%)	2 (33.3%)	1 (16.7%)
	II	-	2 (33.3%)	1 (16.7%)	1 (16.7%)
	III		2 (33.3%)	-	-

0: absence of lesion; I: mild; II: moderate; and III: severe. Number of animals in each group is 6. Numbers 1, 2, 3, 4, 5, and 6 represent the number of animals for each grade. The % in parentheses is the percent of animals in each grade.

## Data Availability

The original contributions presented in this study are included in the article. Further inquiries can be directed to the corresponding authors.
